# Regulation of PD-L1 expression in non–small cell lung cancer by interleukin-1β

**DOI:** 10.3389/fimmu.2023.1192861

**Published:** 2023-06-27

**Authors:** Aiko Hirayama, Kentaro Tanaka, Hirono Tsutsumi, Takayuki Nakanishi, Sho Yamashita, Shun Mizusaki, Yumiko Ishii, Keiichi Ota, Yasuto Yoneshima, Eiji Iwama, Isamu Okamoto

**Affiliations:** Department of Respiratory Medicine, Graduate School of Medical Sciences, Kyushu University, Fukuoka, Japan

**Keywords:** tumor immunology, non-small cell lung cancer, interleukin-1β (IL-1β), mitogen-activated protein kinase (MAPK) pathway, programmed cell death-ligand 1 (PD-L1), interferon-gamma (IFN-γ)

## Abstract

**Introduction:**

Programmed cell death–ligand 1 (PD-L1) is a biomarker for prediction of the clinical efficacy of immune checkpoint inhibitors in various cancer types. The role of cytokines in regulation of PD-L1 expression in tumor cells has not been fully characterized, however. Here we show that interleukin-1β (IL-1β) plays a key role in regulation of PD-L1 expression in non–small cell lung cancer (NSCLC).

**Methods:**

We performed comprehensive screening of cytokine gene expression in NSCLC tissue using available single-cell RNA-Sequence data. Then we examined the role of IL-1β *in vitro* to elucidate its induction of PD-L1 on NSCLC cells.

**Results:**

The IL-1β gene is highly expressed in the tumor microenvironment, particularly in macrophages. The combination of IL-1β and interferon-γ (IFN-γ) induced a synergistic increase in PD-L1 expression in NSCLC cell lines. IL-1β and IFN-γ also cooperatively activated mitogen-activated protein kinase (MAPK) signaling and promoted the binding of downstream transcription factors to the PD-L1 gene promoter. Furthermore, inhibitors of MAPK signaling blocked upregulation of PD-L1 by IL-1β and IFN-γ.

**Discussion:**

Our study reports high levels of IL-1β in the tumor microenvironment may cooperate with IFN-γ to induce maximal PD-L1 expression in tumor cells *via* activation of MAPK signaling, with the IL-1β–MAPK axis being a promising therapeutic target for attenuation of PD-L1–mediated suppression of antitumor immunity.

## Introduction

Immune checkpoint inhibitors (ICIs) have greatly changed the treatment landscape for various cancer types (1–4). Among multiple immune checkpoints that have been identified, agents targeted to that mediated by programmed cell death–1 (PD-1) and its ligand PD-L1 are most effective, and various studies have been undertaken to optimize their efficacy through biomarker-based patient selection or the development of new combination therapies.

Suppression of antitumor immunity in the tumor microenvironment (TME) by immune checkpoints, and in particular that mediated by the PD-1–PD-L1 axis, is a major and common cause of the development of many cancers. Upregulation of PD-L1 expression on tumor cells is thus thought to initiate PD-1–mediated trans-suppression of tumor-specific CD8^+^ T cells. It is therefore important to elucidate the mechanisms responsible for this upregulation of PD-L1 expression. Various such mechanisms have been implicated including those dependent on cytokines, PD-L1 modifiers, signal transduction pathways, microRNAs, and genetic mutations (5). Given that much of the evidence for the operation of such mechanisms has been derived from preclinical models, and the cells studied have included not only tumor cells but various other cell types including endothelial cells and immune cells, the clinical relevance of such findings has remained unknown. Comprehensive studies of human cancer tissue that provide a basis for clinical application of research findings are therefore needed.

Interferon-γ (IFN-γ), a key cytokine produced by effector immune cells such as T lymphocytes and natural killer (NK) cells, has been found to be essential for the upregulation of PD-L1 on tumor cells such as colon cancer, melanoma, and lung cancer cells (5, 6). We therefore hypothesized that, in non–small cell lung cancer (NSCLC), a representative cancer in which many immune cell types contribute to tumorigenesis (7), cytokines produced by such cells might regulate PD-L1 expression on tumor cells in cooperation with IFN-γ. With the use of published data from single-cell analyses, we now show that interleukin (IL–1β) is the most highly expressed cytokine at the transcript level in the TME of NSCLC. We further show that IL-1β stimulation increases the expression of PD-L1 in NSCLC cells in the presence of IFN-γ, and that activation of mitogen-activated protein kinase (MAPK) signaling mediates this upregulation. Our results thus implicate IL-1β as well as MAPK signaling as new targets for immunotherapy in NSCLC.

## Materials and methods

### Cell culture and reagents

A549, H1437, H1373, H838, H23, H322, HCC827, H1650, and H1975 cells were obtained from American Type Culture Collection, PC9 cells from European Collection of Authenticated Cell Cultures, and II-18 cells from RIKEN BRC cell bank. Human monocytic THP-1 cells were kindly provided by Y. Mori and K. Akashi (Kyushu University). These cell lines were cultured in RPMI 1640 medium (Gibco) or Dulbecco’s modified Eagle’s medium (Gibco), each supplemented with 10% fetal bovine serum and 1% penicillin-streptomycin. Phorbol 12-myristate 13-acetate (PMA) (#S7791) was obtained from Selleckchem, and dissolved in dimethyl sulfoxide. Recombinant human IL-2 (R&D Systems, #202-IL) was dissolved in sterile deionized water. Recombinant human IL-1β (R&D Systems, #201-LB) and recombinant human IFN-γ (Peprotech, #300-02) were dissolved in phosphate-buffered saline (PBS) containing 0.1% bovine serum albumin. Fludarabine (#S1491), BAY 11-7082 (#S2913), SP600125 (#S1460), and U0126 (#S1102) were obtained from Selleckchem, and SB203580 was from Invivogen (#tlrl-sb20). Each inhibitor was dissolved in dimethyl sulfoxide. The concentration of each inhibitor was determined based on product data sheets and previous reports (8, 9).

### Gene correlation analysis

**Figure 1:**
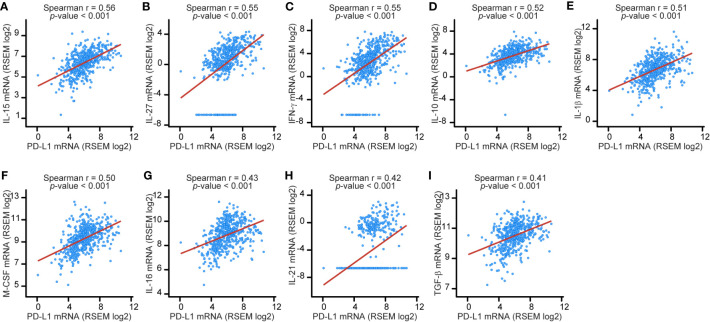
Expression of nine cytokine genes shows a moderate correlation with PD-L1 gene expression in LUAD tissue specimens. The relation between IL-15 **(A)**, IL-27 **(B)**, IFN-γ **(C)**, IL-10 **(D)**, IL-1β **(E)**, M-CSF **(F)**, IL-16 **(G)**, IL-21 **(H)**, or TGF-β **(I)** gene expression and PD-L1 gene expression was examined by Spearman correlation analysis of data from 566 LUAD specimens accessed through cBioportal. The red lines represent regression lines.

The correlation data between cytokine and PD-L1 mRNA in **Figure 1** and **Supplemental Figure S1**, we obtained from cBioportal for cancer genomics data (https://www.cbioportal.org/), an open-access resource. Spearman correlation rank for each comparison is shown and significance is shown with *p*-value.

### Sequencing data analysis

**Figure 2:**
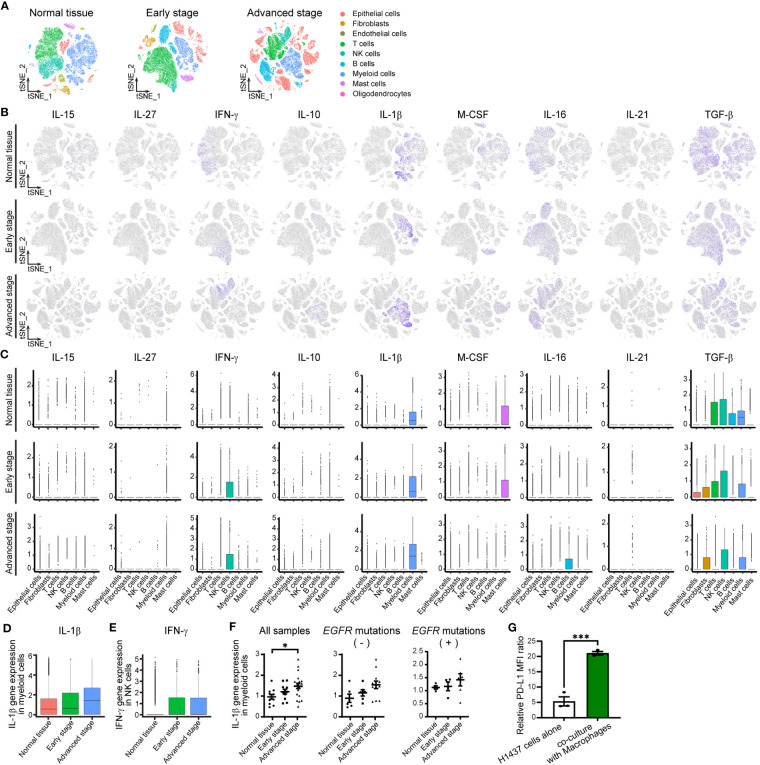
IL-1βmRNA is one of most abundant cytokine mRNAs in the TME of NSCLC. **(A)** tSNE plots for all cells in normal lung tissue (n = 42,995 cells) or early-stage (n = 45,149 cells) or advanced-stage (n = 62,612 cells) NSCLC tissue in an scRNA-seq data set. The plots are color-coded according to cell subsets as indicated. **(B)** Normalized expression of IL-15, IL- 27, IFN-γ, IL-10, IL-1β, M-CSF, IL-16, IL-21, and TGF-β genes shown on the tSNE plots for normal lung tissue (top) or early-stage (middle) or advanced-stage (bottom) tumor tissue. **(C)** Box plots of normalized cytokine gene expression in the indicated cell types of normal lung tissue (top) or early-stage (middle) or advanced-stage (bottom) tumor tissue. Each box represents the median and interquartile range. **(D, E)** Box plots for the normalized abundance of IL-1β mRNA in myeloid (CD68-expressing) cells **(D)** or of IFN-γ mRNA in NK cells **(E)** of normal lung tissue or early-stage or advanced-stage tumor tissue. **(F)** Dot plots of average expression of the IL-1β gene in myeloid cells for each sample of normal tissue (n = 11) or early-stage (n = 11) or advanced-stage (n = 21) tumor tissue. These samples include negative status (normal lung tissue (n = 6) or early-stage (n = 6) or advanced-stage (n = 12) tumor tissue) and positive status (normal lung tissue (n = 4) or early-stage (n = 4) or advanced-stage (n = 6) tumor tissue) for activating *EGFR* mutations. The mean ± SEM values are indicated. **(G)** PD-L1 expression in H1437 cells co-cultured with or without macrophages differentiated by PMA 300 nM. Data are expressed as the relative mean fluorescence intensity (MFI) ratio (PD-L1 to isotype control ratio) and are means ± SEM of triplicates from one experiment that is representative of a total of three independent experiments. *P < 0.05 by the Tukey-Kramer test **(F)** or Student’s *t* test **(G)**.

The raw unique molecular identifier (UMI) count files for the data analyzed in **Figure 2** were obtained from the GEO database (GSE131907). For each tissue data set, the UMI count for the genes in each cell was log-normalized with the NormalizeData function in the Seurat package (v3.2.1) (10) of R software (v4.0.2). The nLung data were analyzed as normal tissue, the tLung data as early-stage tumor tissue, and the mBrain, mLN, and tL/B data were merged for analysis as advanced-stage tumor tissue. Principal components were computed and tSNE clustering was performed with the RunTSNE and FindClusters functions of Seurat. The annotation of each cluster followed the marker genes defined by Kim et al. (11), and M1 and M2 signature genes were curated from a previous study (12). Dot and box plots were generated from log-normalized data. The tSNE clustering results for the data analyzed in **Figures S2A–C** were loaded from “Rdata” files provided on the corresponding github repository (https://github.com/czbiohub/scell_lung_adenocarcinoma). The annotation of each cluster followed the marker genes defined by Maynard et al. (13).

### Differentiation of macrophages and co-culture experiment

THP-1 cells were seeded at 1 × 10^6^ cells/ml, stimulated with 100, 200, or 300 nM PMA for 48 h, and then incubated in fresh RPMI 1640 medium for 72 h to differentiate into THP-1-derived macrophages. The mature phenotype of THP-1-derived macrophages was identified by detection of CD11b using flow cytometry. For the direct co-culture model, THP-1-derived macrophages (2×10^5^ cells) and H1437 cells (1×10^5^ cells) seeded together in a ratio of 2: 1 in 24-well plates for 48 h. Then, cells were harvested for flow cytometry analysis to measure the expression of PD-L1 in H1437 cells.

### Immunoblot analysis

Cells were washed with ice-cold PBS and then lysed in RIPA buffer (Thermo Fisher Scientific) containing protease and phosphatase inhibitor cocktails for preparation of whole cell lysates. A nuclear extract was prepared with the use of NE-PER nuclear and cytoplasmic extraction reagents (Thermo Fisher Scientific). All samples were fractionated by SDS-polyacrylamide gel electrophoresis on a 10% gel, and the separated proteins were transferred to a polyvinylidene difluoride membrane. The membrane was incubated overnight at 4°C with primary antibodies to PD-L1 (#13684), to β-actin (#4970), to phospho-STAT1 (Tyr701) (#7649), to phospho-STAT1 (Ser727) (#9177), to STAT1 (#9172), to phospho–NF-κB p65 (#3033), to NF-κB p65 (#8242), to phospho-p38 (#9211), to p38 (#9212), to phospho-JNK (#4668), to JNK (#9252), to phospho-ERK1/2 (#4370), to ERK1/2 (#9102), to IRF1 (#8478), or to lamin B1 (#12586), all of which were obtained from Cell Signaling Technology (CST). The membrane was subsequently incubated for 1 h at room temperature with horseradish peroxidase–conjugated secondary antibodies to rabbit (Cytivia, #NA9340), after which immune complexes were detected with the use of Pierce ECL Plus Western Blotting Substrate (Thermo Fisher Scientific) or a SuperSignal West Dura Extended Duration Substrate Kit (Thermo Fisher Scientific). Blot images were captured with a ChemiDoc XRS+ system (Bio-Rad).

### RT-qPCR analysis

Total RNA was extracted from cells with the use of a RNeasy Mini Kit (Qiagen) and was subjected to RT with the use of PrimeScript RT Master Mix (Takara). The resulting cDNA was subjected to qPCR analysis with the use of TB Green Premix Ex Taq II (Takara) and a Thermal Cycler Dice Real Time System (Takara). The qPCR primers (forward and reverse, respectively) included those for 18S rRNA (5’-ACTCAACACGGGAAACCTCA-3’ and 5’-AACCAGACAAATCGCTCCAC-3’), IL-1β (5’-TGATGGCTTATTACAGTGGCA-3’ and 5’-GGTCGGAGATTCGTAGCTGG-3’), PD-L1 (5’-CAATGTGACCAGCACACTGAGAA-3’ and 5’-GGCATAATAAGATGGCTCCCAGAA-3’), ERK1 (5’-CTACACGCAGTTGCAGTACAT-3’ and 5’-CAGCAGGATCTGGATCTCCC-3’), and ERK2 (5’-TCACACAGGGTTCCTGACAGA-3’ and 5’-ATGCAGCCTACAGACCAAATATC-3’). The amount of each mRNA was normalized by that of 18S rRNA.

### Flow cytometric analysis

The surface expression of CD326, PD-L1, IL-1R1, IFNGR1, and IFNGR2 in NSCLC cells, and the surface expression of CD11b in THP-1-derived macrophages was quantified by flow cytometry. Cells were stained with antibodies to CD326 (BioLegend, #324306), CD274 (BioLegend, #329708), IL-1R1 (R&D Systems, #FAB269P), IFNGR1 (R&D Systems, #FAB673P), IFNGR2 (R&D Systems, #FAB773A), CD11b (eBioscience, #11-0118-42), allophycocyanin-conjugated mouse IgG2b isotype control (BioLegend, #400322), phycoerythrin-conjugated goat IgG isotype control (BioLegend, #403004), phycoerythrin-conjugated mouse IgG1 isotype control (BioLegend, #400114), allophycocyanin-conjugated goat IgG isotype control (R&D Systems, #IC108A), or fluorescein isothiocyanate-conjugated mouse IgG1 isotype control (BioLegend #400109). For the functional analysis of PBMCs co-cultured with tumor cells, PBMCs were stained with antibody to CD3 (BioLegend, #344833), CD8 (BioLegend, #344711), CD4 (BioLegend, #564420), PD-1 (BioLegend, #329905), or Tim3 (BioLeged, #345011). All samples were analyzed by flow cytometry using a FACSVerse instrument (BD Biosciences). The data were analyzed by Flowjo (v10.5.3) Software. The relative mean fluorescence intensity (MFI) ratio was calculated as the MFI for PD-L1, IL-1R1, IFNGR1, or IFNGR2 divided by that for the corresponding isotype control.

### Isolation and activation of peripheral blood mononuclear cells

Human PBMCs were isolated from peripheral blood of healthy volunteers using BD Vacutainer Mononuclear Cell Preparation Tubes. The acquired PBMCs were cultured in AIM-V medium (Gibco) supplemented with 10% heat-inactivated human male AB plasma (Sigma-Aldrich). Cells were stimulated with Dynabeads coated with anti-CD3 and anti-CD28 monoclonal antibodies (Invitrogen) and IL-2 at 100 IU/ml following the manufacturer’s instructions. The study protocol was authorized by the Ethics Committee of Kyushu University and Kyushu University Hospital (ethics approval ID: 22343-00). All subjects provided written informed consent in accordance with the principles laid out in the Declaration of Helsinki.

### Co-culture experiments of PBMCs and tumor cells

For the functional analysis of PBMCs co-cultured with tumor cells, II-18 cells were preincubated for 24 h in the presence or absence of IL-1β (50 ng/ml). Activated PBMCs were plated in 24-well plates and then co-culture with II-18 cells at 2.5: 1 ratio at 5% CO2, 37°C. After 24 h of co-culture, cells were harvested for flow cytometry. T cells were identified with in CD3^+^ cells and analyzed the expression of CD4 and CD8. And then PD-1 and Tim3 cell surface expression in CD8^+^ T cells was analyzed. For cellular cytotoxicity evaluation, II-18 cells were used at target cells, with or without 24 h pre-treatment by IL-1β (50 ng/ml). Activated PBMCs were washed with PBS, resuspended in fresh media and directly co-cultured with II-18 cells in a 96-well plate at several effector-to-target (E: T) ratios. The plate was incubated at 5% CO2, 37°C for 24 h. The amount of lactate dehydrogenase (LDH) in the supernatant of the co-culture system was detected with the CytoTox96 nonradioactive assay (Promega) following the manufacturer’s instructions. The percent cytotoxicity was calculated as follows: % cytotoxicity = ((experimental – effector spontaneous – target spontaneous) / (target maximum – target spontaneous)) × 100.

### ChIP-qPCR analysis

The binding sites for NF-κB p65, STAT1, IRF1, c-Fos, and Elk1 in the promoter region of the human PD-L1 gene were predicted on the basis of previous studies (14–16) and the JASPAR database (http://jaspar.genereg.net). Chromatin was isolated from H1437 cells with the use of a ChIP Assay Kit (Millipore, #17-295) according to protocols described previously (17). In brief, the cells were first subjected to cross-linking of protein and DNA by exposure to 4% formaldehyde for 10 min at 37°C and ultrasonic treatment, and the DNA-protein complexes were then isolated with the use of the assay kit and with antibodies to NF-κB p65 (CST, #8242), to STAT1 (CST, #9172), to IRF1 (CST, #8478), to c-Fos (CST, #2250), or to Elk1 (CST, #9182), with normal rabbit IgG (CST, #2729) serving as a negative control. DNA was purified with the use of QIA Quick PCR Purification Kit (Qiagen, #28104) and was quantified by real-time PCR analysis with TB Green Premix Ex Taq II (Takara) and a Thermal Cycler Dice Real Time System (Takara). The enrichment of the proteins of interest at the corresponding genomic regions was assessed relative to input DNA. The qPCR primers (forward and reverse, respectively) were 5’-GCAAATCACTGAGCAGCAAG-3’ and 5’-TGGGGATGGGTATTTTGTTT-3’ for NF-κB p65, 5’-AAACTCTTCCCGGTGAAAATC-3’ and 5’-TTGGTGTCCTAGGAATAAAGCTG-3’ for STAT1, 5’-TTCCCGGTGAAAATCTCATT-3’ and 5’-GGCGGAAGCTTTCAGTTTAG-3’ for IRF1, 5’-AAGTTCAGCGCGGGATAATA-3’ and 5’-GTTAGTGAATGGGCCCAAGA-3’ for c-Fos, and 5’-TCTTGGGCCCATTCACTAAC-3’ and 5’-CCTGATATTCTGCCACCCTAA-3’ for Elk1.

### Transfection of small interfering RNA

H1437 cells were plated in 24-well plates and transient transfection with small interfering RNAs (siRNAs) mixed with the RNAiMAX reagent (Thermo Fisher Scientific) at the same time. The siRNAs specific for ERK1 (Thermo Fisher Scientific) and ERK2 (Thermo Fisher Scientific), as well as negative control siRNA (Japan Bio Services). After 48 h of transfection, H1437 cells were treated with or without both IL-1β and IFN-γ for 24 h. Then the cells were harvested and subjected to real-time RT-PCR.

### Statistical analysis

Statistical analysis was performed with GraphPad Prism 8. Data were compared between two groups with the unpaired Student’s *t* test or among more than two groups with the Tukey-Kramer test. A *p*-value of <0.05 was considered statistically significant.

## Results

### IL-1β mRNA is the most abundant cytokine mRNA and its amount is significantly correlated with that of PD-L1 mRNA in the TME of NSCLC

To identify cytokines important for the regulation of PD-L1 expression in NSCLC, we first comprehensively examined the relation between expression of the genes for 26 major cytokines and PD-L1 gene expression in lung adenocarcinoma (LUAD) with the use of data from 566 LUAD tissue specimens available through cBioportal for cancer genomics data (**Supplementary Table 1**). We found that the expression of nine cytokine genes showed a moderate positive correlation with PD-L1 gene expression (**Figure 1**) (18). Expression of the remaining cytokine genes showed a weak or negligible correlation with PD-L1 gene expression (**Supplementary Figure S1**).

To confirm the expression and identify the source of these nine cytokines in the TME of human NSCLC, we analyzed two different published data sets obtained by scRNA-seq analysis (11, 13). Assessment of the expression of the cytokine genes in 23,261 cells in one of these data sets (13) by t-distributed stochastic neighbor embedding (tSNE) revealed that expression of the IL-1β gene in subsets of macrophages and monocytes was the most pronounced in the TME of NSCLC (**Supplementary Figures S2A–C**; **Tables 2, 3**). We then analyzed the second data set for 150,756 cells (11) classified into nine distinct cell lineages including epithelial, stromal, and immune cells as well as according to disease stage, specifically as normal tissue, early stage, or advanced stage (**Figure 2A**; **Supplementary Tables 4, 5**). Consistent with our findings for the first data set, IL-1β gene expression was prominent in myeloid cells, whereas substantial expression was also apparent for the IFN-γ gene in NK cells, the macrophage colony-stimulating factor (M-CSF) gene in mast cells, and the transforming growth factor–β (TGF-β) gene in various cell types (**Figures 2A–C**). The genes for the other cytokines were poorly expressed. Analysis of IL-1β gene expression in myeloid cells and IFN-γ gene expression in NK cells according to disease stage revealed that the expression of both genes tended to be increased in tumor tissue compared with normal tissue, whereas only IL-1β gene expression tended to increase with tumor stage progression (**Figures 2D, E**). We also examined IL-1β and IFN-γ gene expression in individual patients and found that IL-1β gene expression in myeloid cells was significantly associated with tumor progression, whereas such an association was not apparent for IFN-γ gene expression in NK cells or T cells (**Figure 2F**; **Supplementary Figures S2D, E**; **Table 6**).

To elucidate the characteristics of myeloid cells or macrophages as a source of IL-1β, we assessed the status of tumor-associated macrophage (TAM) polarization according to the M1-macrophage or M2-macrophage phenotype by examining the expression of respective signature genes (12, 19) (**Supplementary Figure S3**, **Table 7**). Among genes thought to be related to the M1-macrophage phenotype, expression of the IL-8 gene increased with tumor progression together with that of the IL-1β gene. On the other hand, the expression of many M2 signature genes—including those for FN1, MARCO, MRC1, MSR1, TGF-β1, and TGM2—decreased with tumor progression. Macrophages in the TME of advanced NSCLC thus retain M1-like features characterized by high expression of IL-1β and IL-8.

Because macrophages were found to be a major source of IL-1β in the TME of NSCLC, we examined surface PD-L1 expression in H1437 cells co-cultured with macrophages. THP-1 cells were differentiated into macrophages by stimulation with PMA, and which characterized by increased cell surface expression of CD11b (**Supplementary Figure S4A**) (20). Reverse transcription (RT)–quantitative polymerase chain reaction (qPCR) analyses revealed that PMA stimulation increased the amount of IL-1β mRNA in macrophages (**Supplementary Figure S4B**). Then, H1437 cells were co-cultured with macrophages differentiated by PMA 300 nM stimulation. The results showed that macrophages induced expression of PD-L1 in H1437 cells (**Figure 2G**; **Supplementary Figure S4C**).

### Effect of IL-1β on PD-L1 expression in NSCLC cells

**Figure 3:**
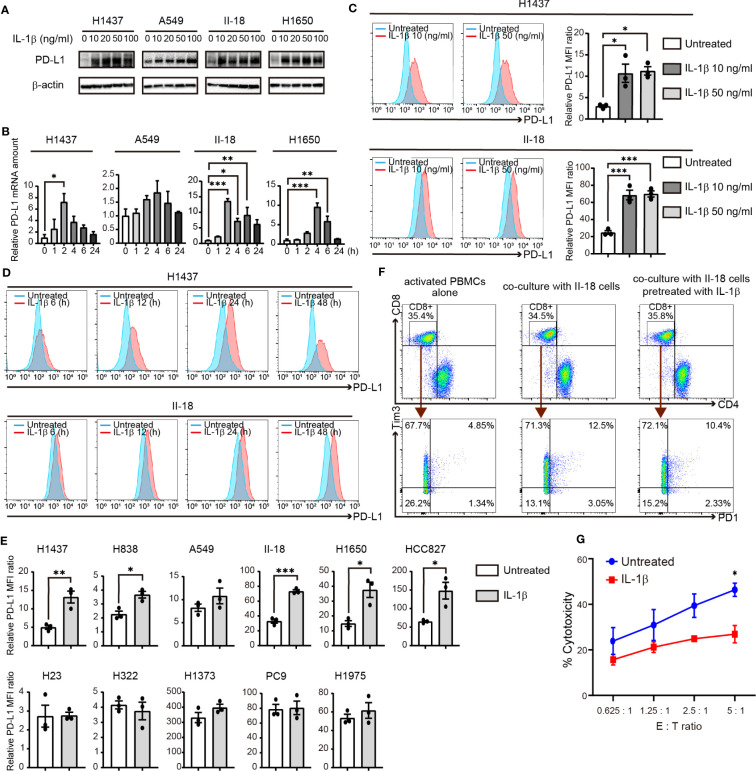
IL-1β induces the expression of PD-L1 in NSCLC cells. **(A)** Immunoblot analysis of PD-L1 and β-actin (loading control) in H1437, A549, II-18, and H1650 cells treated with various concentrations of IL-1β for 24 h. **(B)** RT-qPCR analysis of PD-L1 mRNA abundance in H1437, A549, II-18, and H1650 cells treated with IL-1β (50 ng/ml) for various times. Data are expressed relative to the value for time 0 and are means + SEM of triplicates from one experiment that is representative of a total of three independent experiments. **(C)** Flow cytometric analysis of surface PD-L1 expression in H1437 and II-18 cells treated with IL-1β at 10 or 50 ng/ml for 24 h. Representative traces and quantitative data are shown, with the latter being expressed as the relative MFI ratio (PD-L1 to isotype control ratio) and presented as means ± SEM from three independent experiments. **(D)** Flow cytometric analysis of surface PD-L1 expression in H1437 and II-18 cells treated with IL-1β at 50 ng/ml for 6, 12, 24, or 48 h. **(E)** Quantitative flow cytometric determination of surface PD-L1 expression in the indicated NSCLC cell lines treated (or not) with IL-1β (50 ng/ml) for 24 h. Data are expressed as the relative MFI ratio (PD-L1 to isotype control ratio) and are means ± SEM from three independent experiments. **(F)** II-18 cells were preincubated for 24h in the presence or absence of IL-1β (50 ng/ml) before co-cultured with activated PBMCs with effector to target ratios of 2.5: 1. Flow cytometric plots to show the expression of PD1 and Tm3 on CD8^+^ T cells from activated PBMCs alone and co-cultured PBMCs with II-18 cells for 24 h. **(G)** Cytotoxicity assay of activated PBMCs (Effector cells) against II-18 cells pretreated with or without IL-1β (50 ng/ml, 24 h) (Target cells). *P < 0.05, **P < 0.01, ***P < 0.001 by the Tukey-Kramer test **(B, C)** or Student’s *t* test **(E, G)**.

We next examined the effect of direct stimulation with IL-1β on PD-L1 expression in human NSCLC cell lines. Immunoblot and RT-qPCR analyses revealed that IL-1β treatment increased the amounts of PD-L1 protein and mRNA in H1437, A549, II-18, and H1650 cells (**Figures 3A, B**). Flow cytometry also showed that IL-1β upregulated the surface expression of PD-L1 in these cell lines (**Figures 3C, D**; **Supplementary Figures S5A, B**). We then examined additional cell lines and categorized all 11 cell lines studied according to their negative (H1437, H838, A549, H23, H322, and H1373) or positive (II-18, H1650, HCC827, PC9, and H1975) status for activating *EGFR* mutations. Quantitative flow cytometric analysis revealed that IL-1β significantly increased or tended to increase PD-L1 expression in 6 of the 11 cell lines examined (H1437, H838, A549, II-18, H1650, and HCC827) with these six cell lines including those both negative or positive for *EGFR* mutations (**Figure 3E**). We also examined the expression of IL-1 receptor type 1 (IL-1R1) in the NSCLC cell lines but found no marked differences between those that responded to IL-1β stimulation and those that did not (**Supplementary Figures S5C, D**). These results thus showed that a substantial proportion of NSCLC cell lines respond to IL-1β stimulation by increasing the expression of PD-L1.

To examine the functional profile of CD8^+^ T cells, we performed co-cultures experiments of activated PBMCs and tumor cells. II-18 cells were pretreated with or without IL-1β for 24 h and then co-cultured with activated PBMCs. Regardless of IL-1β pre-treatment, after 24 h co-culture with II-18 cells, co-expression of PD-1 and Tim3, markers of exhausted T cells, was increased in CD8^+^ T cells (**Figure 3F**) (21). Furthermore, the effect of IL-1β-induced PD-L1 in tumor cells on activated PBMCs was evaluated in cytotoxicity assay. II-18 cells pretreated with IL-1β showed lower lysis rates than II-18 cells non-pretreated (**Figure 3G**).

### Cooperative effect of IL-1β and IFN-γ on PD-L1 expression in NSCLC cells

**Figure 4:**
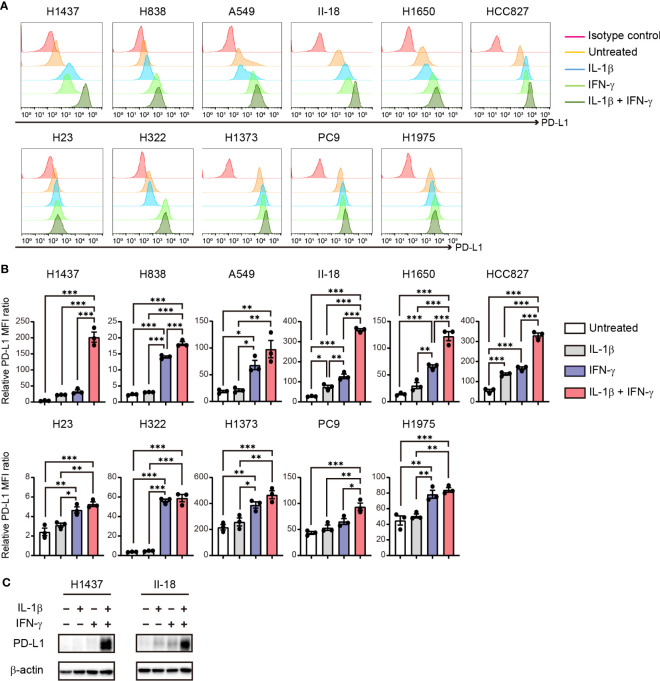
Cooperative effect of IL-1β and IFN-γ on PD-L1 expression in NSCLC cells. **(A, B)** Representative flow cytometric traces for **(A)** and quantitation of **(B)** surface PD-L1 expression in NSCLC cells treated with IL-1β (50 ng/ml, 36 h), IFN-γ (50 ng/ml, 24 h), or the combination of IL-1β (50 ng/ml, 36 h) and IFN-γ (50 ng/ml, 24 h). For the combined treatment, the cells were exposed to IL-1β for 12 h before the addition of IFN-γ. The quantitative data are expressed as relative MFI ratio (PD-L1 to isotype control ratio) and are means ± SEM from three independent experiments. **(C)** Immunoblot analysis of PD-L1 abundance in H1437 and II-18 cells treated with IL-1β (50 ng/ml), IFN-γ (50 ng/ml), or both agents (each at 50 ng/ml) for 24 h. *P < 0.05, **P < 0.01, ***P < 0.001 by the Tukey-Kramer test.

Our findings thus revealed that PD-L1 expression on tumor cells is increased by stimulation with IL-1β, which is produced by macrophages in the TME. In addition, the results of cytotoxicity assay suggested that pre-treatment of tumor cells with IL-1β suppress T cell activity. Previous study has shown that IFN-γ is secreted when tumor cells and activated PBMCs are co-cultured (22). Given that IFN-γ is a well-characterized inducer of PD-L1 expression in NSCLC cells (23), we examined the effects of IL-1β secreted by macrophages and IFN-γ secreted by T cells, alone or together, on PD-L1 expression in NSCLC cell lines *in vitro*. Flow cytometry revealed that, in all cell lines examined, IFN-γ treatment tended to induce a higher level of PD-L1 expression than did IL-1β treatment (**Figures 4A, B**). Combined stimulation with IL-1β and IFN-γ significantly increased PD-L1 expression in all cells compared to untreated or IL-1β alone. And the combination of IL-1β and IFN-γ induced a higher level of PD-L1 expression than did IFN-γ alone in all cell lines with the exception of H322 and H1975, with this difference being significant in H1437, H838, II-18, H1650, HCC827, and PC9 cells (**Figures 4A, B**). Flow cytometric analysis of the IFN-γ receptors IFNGR1 and IFNGR2 in these various cell lines revealed that IL-1β stimulation increased the expression of IFNGR1 in H1437, A549, II-18, and H1650 cells but had no effect on IFNGR2 expression in any of the examined cell lines (**Supplementary Figure S6**), suggesting that an effect of IL-1β on the expression of these receptors did not fully account for the cooperative effect of IL-1β and IFN-γ on PD-L1 expression. Immunoblot analysis of H1437 and II-18 cells showed that this combined effect of the two cytokines on surface expression of PD-L1 reflected an increase in total PD-L1 abundance (**Figure 4C**). Together, these results thus showed that combined stimulation with IFN-γ and IL-1β maximized the upregulation of PD-L1 expression in a substantial proportion of NSCLC cell lines.

### Nuclear IRF1 localization and MAPK activation in NSCLC cells stimulated with IL-1β and IFN-γ

**Figure 5:**
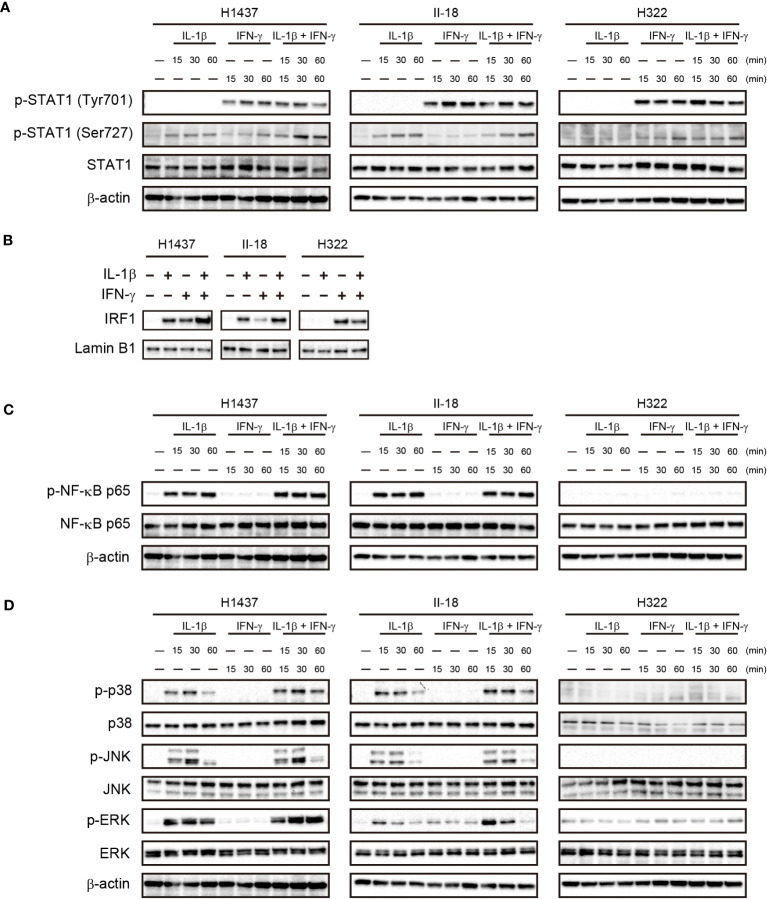
Cooperative effects of IL-1β and IFN-γ on nuclear translocation of IRF1 and ERK activation in association with a synergistic effect on PD-L1 expression. H1437, II-18, and H322 cells were treated with IL-1β (50 ng/ml), IFN-γ (50 ng/ml), or both IL-1β (50 ng/ml) and IFN-γ (50 ng/ml) for the indicated times, after which cell lysates were subjected to immunoblot analysis with antibodies to total or phosphorylated (p) forms of STAT1 **(A)**, the p65 subunit of NK-κB **(C)**, or the MAPKs p38, JNK, and ERK **(D)**. Alternatively, a nuclear fraction prepared from the cells stimulated for 60 min was subjected to immunoblot analysis with antibodies to IRF1 and with those to lamin B1 as a loading control for the nucleus **(B)**.

To investigate the mechanism underlying the cooperative effect of IL-1β and IFN-γ on PD-L1 expression in NSCLC cells, we examined the activation of downstream signaling events by these cytokines in the H1437 and II-18 cells, both of which showed a synergistic effect of IL-1β and IFN-γ on PD-L1 expression, as well as in H322 cells, which showed an effect only of IFN-γ. IFN-γ treatment similarly increased the phosphorylation of STAT1 (signal transducer and activator of transcription 1) (Tyr701) in all three cell lines, whereas IL-1β had no effect on STAT1 (Tyr701) phosphorylation with or without IFN-γ. On the other hand, phosphorylation of STAT1 (Ser727) was increased by the combination of IL-1β and IFN-γ than by IL-1β or IFN-γ alone in H1437 cells, whereas it was unaffected by combination of IL-1β and IFN-γ in II-18 and H322 cells (**Figure 5A**). We next examined the nuclear translocation of IRF1 (interferon regulatory factor 1), a key downstream transcription factor of STAT1. The amount of IRF1 in the nucleus of H1437 or II-18 cells was increased by the combination of IL-1β and IFN-γ to a greater extent than by treatment with either cytokine alone, whereas it was increased by IFN-γ but unaffected by IL-1β in H322 cells (**Figure 5B**). IL-1β increased phosphorylation of the p65 subunit of the transcription factor NF-κB in H1437 and II-18 cells but not in H322 cells, whereas IFN-γ had no effect on p65 phosphorylation with or without IL-1β in any of the cell lines (**Figure 5C**). Analysis of MAPK signaling revealed that IFN-γ alone had no effect on phosphorylation of the MAPKs p38, JNK (c-Jun NH2-terminal kinase), or ERK (extracellular signal–regulated kinase) in any of the three cell lines (**Figure 5D**). However, phosphorylation of ERK was increased to a greater extent by the combination of IL-1β and IFN-γ than by IL-1β alone in H1437 and II-18 cells, whereas it was unaffected by IL-1β with or without IFN-γ in H322 cells (**Figure 5D**). Phosphorylation of p38 or JNK was induced to similar extents by IL-1β with or without IFN-γ in H1437 and II-18 cells, but was unaffected by IL-1β in H322 cells (**Figure 5D**). These various observations thus suggested that the synergistic effect of IL-1β and IFN-γ on PD-L1 expression was associated with IRF1 translocation to the nucleus and activation of MAPK signals such as ERK.

### MAPK activation is indispensable for the synergistic upregulation of PD-L1 expression by IL-1β and IFN-γ in NSCLC cells

**Figure 6:**
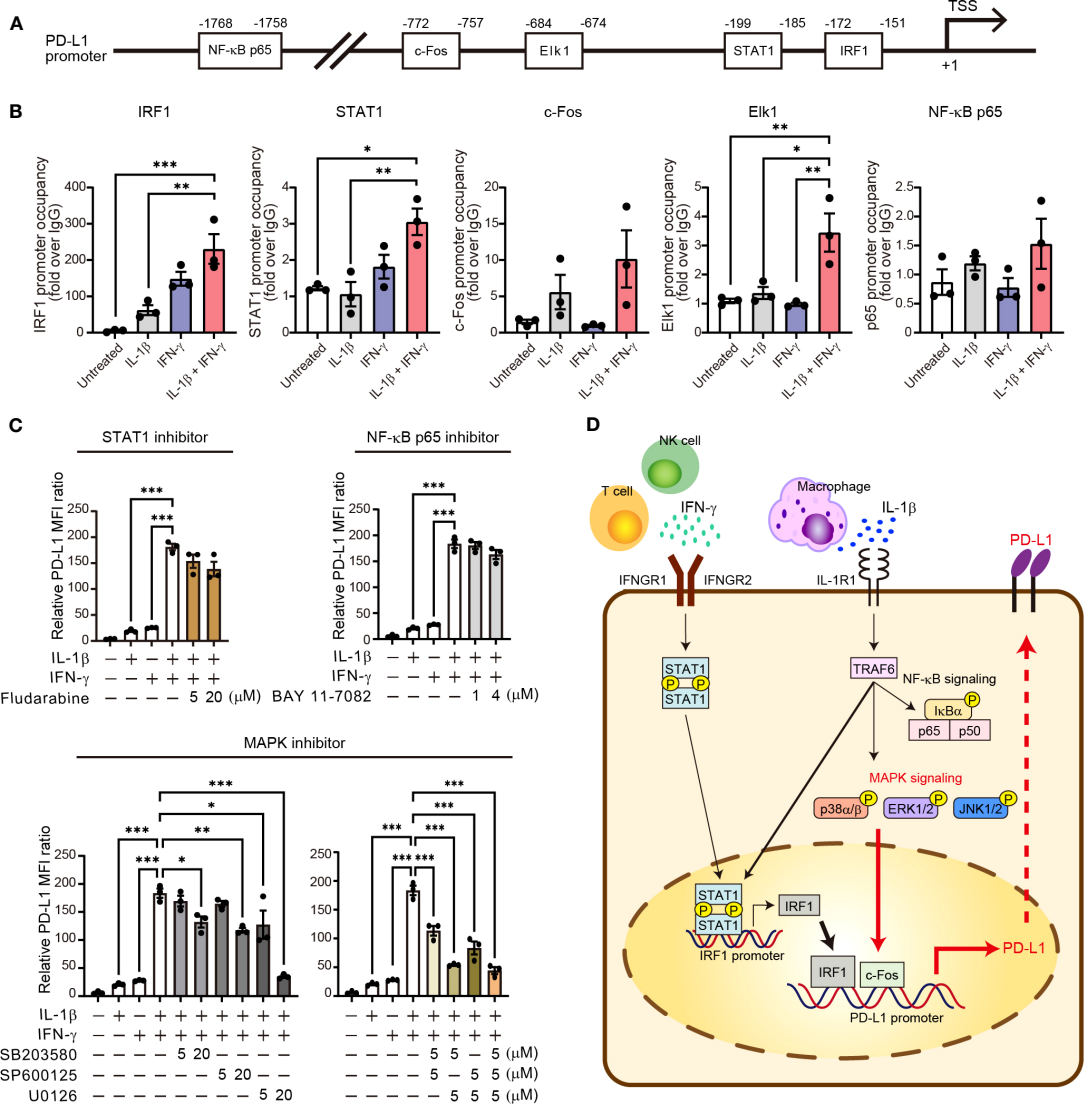
Activation of MAPK signaling is essential for synergistic upregulation of PD-L1 expression by IL-1β and IFN-γ. **(A)** Schematic representation of the promoter region of the human PD-L1 gene showing the transcription start site (TSS) as well as predicted binding sites for NF-κB p65, c-Fos, Elk1, STAT1, and IRF1. **(B)** H1437 cells were treated with IL-1β (50 ng/ml), IFN-γ (50 ng/ml), or both IL-1β (50 ng/ml) and IFN-γ (50 ng/ml) for 2 h and were then subjected to ChIP-qPCR analysis with antibodies to the various transcription factors (or with control immunoglobulin G [IgG]) and with qPCR primers targeted to the corresponding binding regions. Data are expressed relative to the signal obtained with control IgG and are means ± SEM from three independent experiments. **(C)** H1437 cells were incubated for 1 h with or without Fludarabine (5 or 20 μM), BAY 11-7082 (1 or 4 μM), SB203580 (5 or 20 μM), SP600125 (5 or 20 μM), or U0126 (5 or 20 μM) and then for 24 h in the additional absence or presence of IL-1β (50 ng/ml), IFN-γ (50 ng/ml), or both IL-1β (50 ng/ml) and IFN-γ (50 ng/ml), after which surface expression of PD-L1 was determined by flow cytometry. The quantitative data are expressed as relative MFI ratio (PD-L1 to isotype control ratio) and are means ± SEM from three independent experiments. **(D)** Proposed model for the role of IL-1β and IFN-γ signaling in the regulation of PD-L1 expression in NSCLC. IFN-γ derived from T cells and NK cells and IL-1β derived from macrophages together induce transcription of the PD-L1 gene in tumor cells *via* STAT1-IRF1 and MAPK–c-Fos axes. *P < 0.05, **P < 0.01, ***P < 0.001 by the Tukey-Kramer test.

To characterize transcriptional activation of the promoter region of the PD-L1 gene by the combination of IL-1β and IFN-γ, we examined the binding of various downstream transcription factors including STAT1, IRF1, NF-κB p65, c-Fos, and Elk1 in H1437 cells. We searched for potential binding sites in the promoter region of the PD-L1 gene with JASPAR, an open-access database of transcription factor binding profiles (http://jaspar.genereg.net). All of these transcription factors had potential binding sites within the 2-kbp region upstream of the transcription start site (TSS) of the human PD-L1 gene (**Figure 6A**). To confirm the binding of these transcription factors to these sites, we performed chromatin immunoprecipitation (ChIP)–qPCR analysis (**Figure 6B**). We found that binding of IRF1 to the PD-L1 promoter was increased by IL-1β or IFN-γ alone and to a greater extent by the combination of both agents. The binding of STAT1 was increased by IFN-γ and to a greater extent by both IL-1β and IFN-γ, but the magnitude of the latter effect was much less than that apparent for IRF1. Both c-Fos and Elk1 function downstream of MAPK signaling, and we found that c-Fos binding to the PD-L1 promoter tended to be increased by IL-1β alone and to a greater extent by both cytokines, whereas Elk1 binding was specifically and gently increased by combined stimulation with IL-1β and IFN-γ. Neither cytokine had an effect on the binding of the p65 subunit of NF-κB to the PD-L1 gene promoter. Together, these ChIP-qPCR results implicated STAT1, IRF1, and MAPK-targeted transcription factors in the maximal induction of PD-L1 expression by the combination of IL-1β and IFN-γ.

Finally, we examined the effects of specific signaling inhibitors on the synergistic induction of PD-L1 expression by IL-1β and IFN-γ. SB203580 (p38 inhibitor), SP600125 (JNK inhibitor), and U0126 (inhibitor of the ERK kinase MEK) each significantly attenuated in a concentration-dependent manner the increase in PD-L1 expression induced in H1437 cells by combined stimulation with IL-1β and IFN-γ (**Figure 6C**). Indeed, U0126 at 20 µM or the combination of U0126 at 5 µM and either SB203580 or SP600125 at 5 µM almost abolished the synergistic effect of IL-1β and IFN-γ on PD-L1 expression in these cells. In contrast, Fludarabine (STAT1 inhibitor) and BAY 11-7082 (NF-κB p65 inhibitor) had no effect on the induction of PD-L1 expression by the combination of IL-1β and IFN-γ (**Figure 6C**). Similar results were obtained with II-18 cells as with H1437 cells (**Supplementary Figure S7A**), although the various inhibitors also appeared to attenuate cell survival in this cell line. U0126 at 20 μM blocked the phosphorylation of ERK both induced by IL-1β alone and combination of IL-1β and IFN-γ, it did not affect p38, JNK, STAT1, and NF-κB phosphorylation (**Supplementary Figure S7B**). We obtained similar results with siRNA interference assay using the effective siRNA of ERK. The results showed that ERK siRNA suppressed the PD-L1 expression induced by combination of IL-1β and IFN-γ (**Supplementary Figure S7C**). Together, these results suggested that activation of MAPK signaling pathways, especially ERK, is essential for the synergistic induction of PD-L1 expression by combined stimulation with IL-1β and IFN-γ (**Figure 6D**).

## Discussion

We have here shown that the canonical inflammatory cytokine IL-1β present in the TME of NSCLC contributes to the upregulation of PD-L1 expression in tumor cells. Comprehensive analysis of public databases and published data revealed that macrophages are a major source of IL-1β in the TME of NSCLC. Expression of the IL-1β gene in myeloid cells was thus found to be increased in tumor tissue relative to normal lung tissue and to increase with disease progression. We then showed that IL-1β alone upregulates PD-L1 expression in certain NSCLC cell lines, and pre-treatment with IL-1β decreased the cell lysis mediated by lymphocytes. Furthermore, our findings showed that combined stimulation with IFN-γ and IL-1β maximized the upregulation of PD-L1 expression in a substantial proportion of NSCLC cell lines. Finally, our results implicated MAPK and STAT1-IRF1 signaling pathways in this synergistic effect, which was efficiently blocked by MAPK inhibitors. Our findings, together with a recent study showing that air pollutants cause an influx of macrophages into the lung and release of IL-1β (24), demonstrate that macrophage-derived IL-1β in the lung is a pivotal cytokine in the development and progression of lung cancer.

The IL-1 family is composed of 11 soluble molecules that interact with 10 receptors (25). Among these molecules, IL-1β is a well-characterized proinflammatory cytokine that is produced by cells in multiple organs including the gastrointestinal tract and lung in response to various challenges such as bacteria, viruses, dietary factors, and inhaled particulates. Although it functions to initiate appropriate innate and adaptive immune responses, aberrant expression of IL-1β has been implicated in the development of many diseases, including autoimmune conditions, metabolic disorders, and cardiovascular disease. Canakinumab, a human neutralizing monoclonal antibody to IL-1β, has been approved for the treatment of systemic inflammatory diseases such as cryopyrin-associated periodic syndrome (CAPS), systemic-onset juvenile idiopathic arthritis, and refractory gout (26).

With regard to the function of IL-1β in cancer, its expression has been found to be positively associated with tumor promotion in mice (27–29) as well as in humans (24, 30, 31), with effects of IL-1β on various processes including angiogenesis, tumor cell invasion, and immunosuppression having been implicated as underlying this association. Several phase III studies of canakinumab treatment with implications for lung cancer have recently been reported. The Canakinumab Anti-inflammatory Thrombosis Outcome Study (CANTOS, NCT01327846) (32–34) was performed primarily to analyze the outcome of patients with atherosclerosis and myocardial infarction, but an exploratory analysis indicated that treatment with canakinumab reduced the incidence and mortality only of lung cancer among multiple tumors investigated, with this effect appearing to be dose dependent. These data suggested that addition of an IL-1β antibody to standard treatment might improve the outcome of patients with metastatic NSCLC. This notion has been investigated by the CANOPY program consisting of four trials (CANOPY-1, NCT03631199; CANOPY-2, NCT03626545; CANOPY-A, NCT03447769; and CANOPY-N, NCT03968419) (35, 36). However, three of these studies, in which canakinumab was combined with cytotoxic drugs and/or an ICI, did not show a significant survival advantage of canakinumab addition, with combination therapy even showing a tendency to increase severe infection (37–39). The clinical strategy to take advantage of anti-IL-1β blockade in cancers has thus remained undetermined.

We have now revealed an indispensable role for IL-1β in the upregulation of PD-L1 expression in NSCLC cells, suggesting the possibility that IL-1β might increase the incidence and mortality of NSCLC, consistent with the findings of the CANTOS study. Blockade of IL-1β activity by administration of canakinumab might thus be expected to antagonize the upregulation of PD-L1 on tumor cells. Infiltration of tumor-specific CD8^+^ T cells and NK cells into the TME and their production of IFN-γ might therefore promote the elimination of tumor cells by immune cells under canakinumab treatment, instead of promoting PD-L1–mediated immune suppression in combination with endogenous IL-1β in the TME. Our results based on scRNA-seq analysis and *in vitro* experiments suggest that IL-1β in the TME of NSCLC is derived largely from TAMs. These cells have been thought to control tumor progression through various effects, including regulation of angiogenesis and differentiation of cancer stem cells as well as modification of immunity (40), similar to the reported functions of IL-1β. And, our results showed that IL-1β produced by macrophages induced PD-L1 expression in NSCLC cell lines. We found that increased expression of the IL-1β gene in TAMs of advanced NSCLC was associated with high expression of the IL-8 gene. This finding based on scRNA-seq analysis is consistent with the previous observations that IL-1β was expressed in IL-8^high^ myeloid cells and that high IL-8 expression was associated with a reduced clinical benefit of PD-1 blockade therapy for metastatic urothelial carcinoma or renal cell carcinoma (41). High expression of IL-8 has also been associated with tumorigenesis, tumor progression, and suppression of the antitumor immune response in various types of cancer (42, 43). Increased expression of IL-1β associated with high IL-8 expression in macrophages might therefore contribute to the function of TAMs as a key tumor promoter in the TME. Given that patients with substantially increased serum levels of C-reactive protein and IL-6, direct downstream inflammatory effectors of IL-1β, were eligible for the CANTOS trial, it is possible that clinical development of IL-1β–targeted agents in combination with ICIs might improve outcome in NSCLC patients with high expression of IL-1β as well as IL-8 in tumor tissue.

Our results now suggest that MAPK signaling is a potential new therapeutic target to inhibit pathogenic expression of PD-L1 on tumor cells induced by combined stimulation with IFN-γ and IL-1β. Previous studies have shown that PD-L1 expression in unstimulated cells can be suppressed by MAPK inhibitors, and that such inhibitors in combination with PD-1 blockade can efficiently attenuate tumor growth in preclinical models (8, 44), suggesting that MAPK signaling is a key regulator of PD-L1 expression as well as of antitumor immunity. We have now shown that combined stimulation of NSCLC cells with IL-1β and IFN-γ induced both MAPK activation and recruitment of downstream transcription factors such as c-Fos to the promoter region of the PD-L1 gene. A functional role of MAPK signaling in upregulation of PD-L1 expression by IL-1β and IFN-γ was demonstrated by our observation that such upregulation was attenuated by inhibitors of such signaling. This MAPK signaling may cooperate with recruitment of IRF1 to the PD-L1 gene promoter to mediate upregulation of PD-L1 expression by these cytokines. Previous studies suggested the crosstalk between IFN-γ–STAT1–IRF1 and IL-1β–MAPK–c-Fos signaling *via* an adaptor protein, myeloid differentiation factor 88 (MyD88) (45, 46). Basal stimulation of IL-1β in the TME could activate MyD88 to increase the transcription of IFN-γ downstream genes. Further studies of the relative roles of and interactions in these pathways in the upregulation of PD-L1 expression in tumor cells are warranted.

There are several limitations to our study. First, we do not have any *in vivo* evidence of a direct relation between pathogenic PD-L1 expression induced by IL-1β and IFN-γ and actual tumor development. Second, the association of IL-1β with pathogenic PD-L1 regulation functions consistently regardless of *EGFR* gene status, while the proportion of NSCLC tumors with high PD-L1 expression tends to be lower for patients with activating *EGFR* mutations than for those wild type for *EGFR* (47), with mechanisms associated with such mutations appearing to negatively regulate PD-L1 expression. Third, how some cells such as H322 cells were completely unresponsive to IL-1β stimulation cannot be examined. Finally, the mechanism by which combined stimulation with IL-1β and IFN-γ specifically activates MAPK signaling in tumor cells remains unknown.

Substantial issues regarding ICI treatment for advanced cancer have emerged, including a limited response and inevitable development of treatment resistance (48). New approaches are thus needed to overcome these issues. Our results showing the importance of IL-1β as a regulator of pathogenic PD-L1 expression in tumor cells may provide a scientific basis for further understanding of PD-L1–PD-1 axis signaling and for development of novel agents to inhibit PD-L1 expression and thereby to promote the immune-mediated elimination of tumors.

## Data availability statement

The original contributions presented in the study are included in the article/**Supplementary Material**. Further inquiries can be directed to the corresponding author.

## Author contributions

AH and KT designed the study and contributed to writing of the manuscript. KT and IO obtained funding. AH performed all experiments with assistance from HT, TN, and SY. SM and YI supported co-culture experiments tumor cells with immune cells. AH, KT, KO, YY, EI, and IO contributed to data analysis and interpretation. All authors contributed to the article and approved the submitted version.
