# Supplementary material for: Regulation of PD-L1 expression in non–small cell lung cancer by interleukin-1β
Source: Front Immunol. 2023 Jun 27;14:1192861. doi: 10.3389/fimmu.2023.1192861 (PMC10333574; doi:10.3389/fimmu.2023.1192861)
Supplement: Supplementary file 2 [file DataSheet_1.docx]

Supplementary Material

**Regulation of PD-L1 expression in non–small cell lung cancer by interleukin-1β**

**Aiko Hirayama^1, †^, Kentaro Tanaka^1, †, *^, Hirono Tsutsumi^1^, Takayuki Nakanishi^1^, Sho Yamashita^1^, Shun Mizusaki^1^, Yumiko Ishii^1^, Keiichi Ota^1^, Yasuto Yoneshima^1^, Eiji Iwama^1^, Isamu Okamoto^1^**

^1^Department of Respiratory Medicine, Graduate School of Medical Sciences, Kyushu University; Fukuoka, Japan

† These authors contributed equally to this work.

*** Correspondence:**Kentaro Tanaka
Department of Respiratory Medicine, Graduate School of Medical Sciences, Kyushu University

3-1-1 Maidashi, Higashi-ku, Fukuoka, 812-8582, Japan

Phone: +81-92-642-5378, Fax: +81-92-642-5382

E-mail address: tanaka.kentaro.983@m.kyushu-u.ac.jp


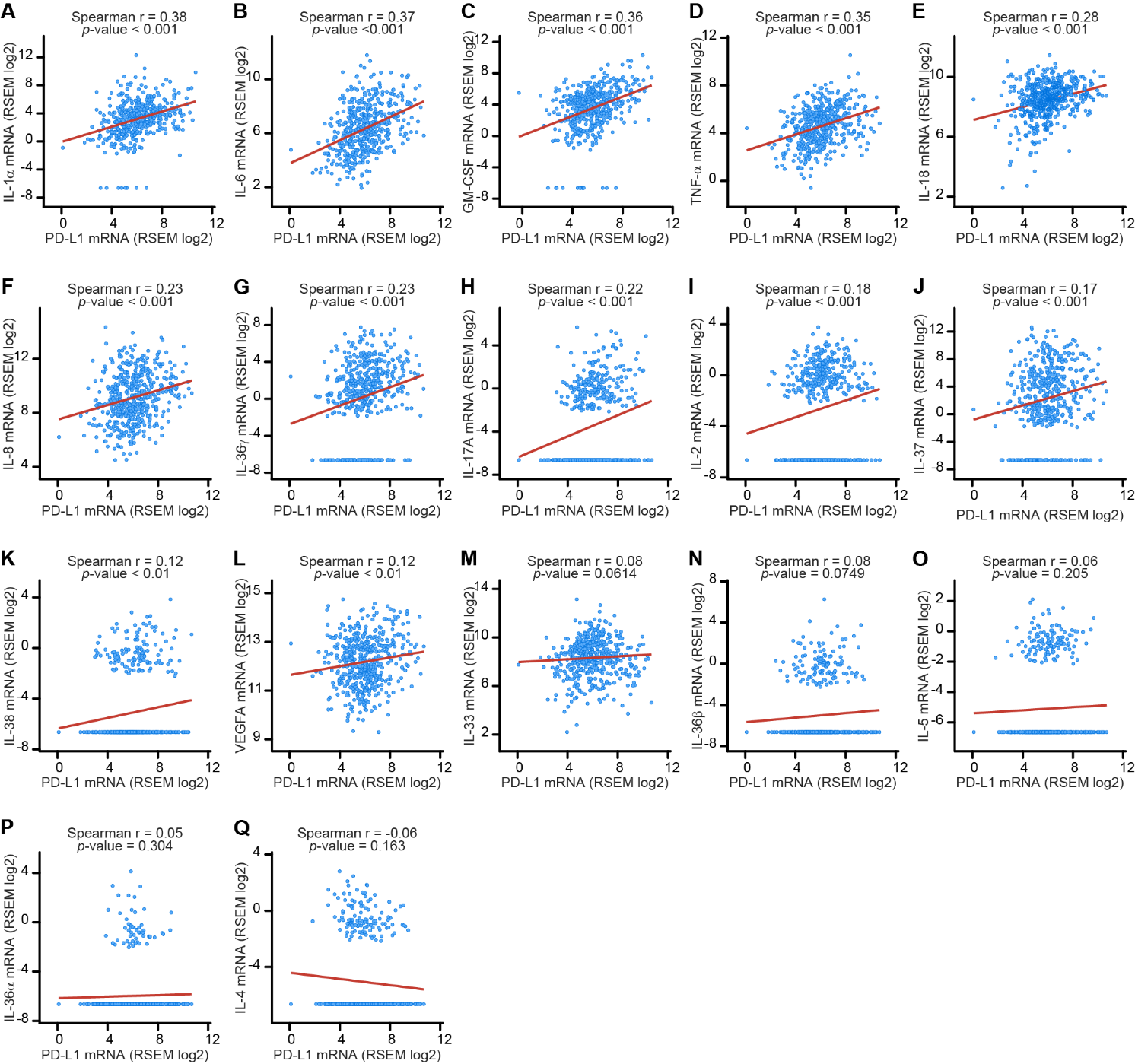


**Supplementary Figure 1. Correlation analysis for cytokine and PD-L1 mRNA abundance in LUAD.** The relation between expression of the indicated cytokine genes and PD-L1 gene expression was examined by Spearman correlation analysis of data from 566 LUAD specimens accessed through cBioportal. The red lines represent regression lines.


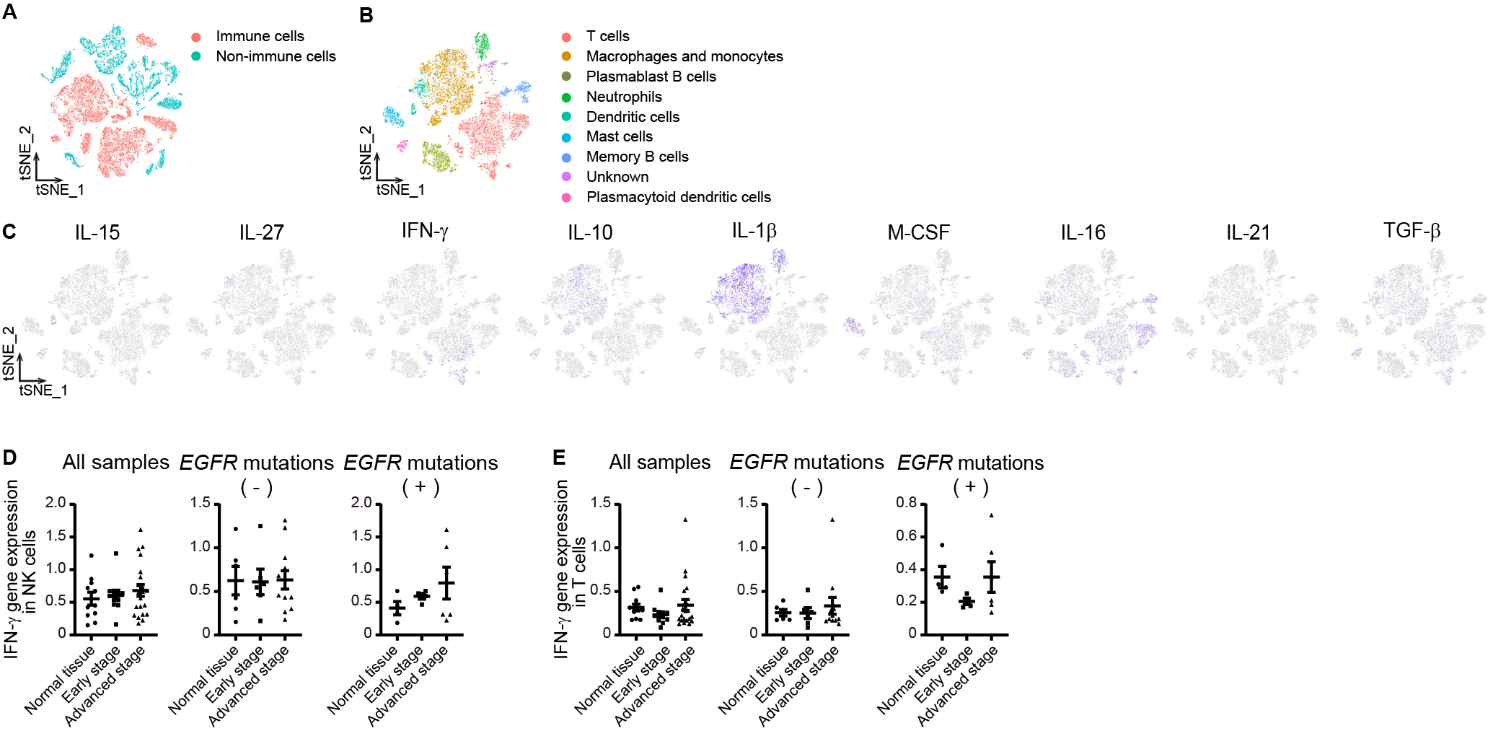


**Supplementary Figure 2. IL-1β mRNA is abundant in the TME of NSCLC.** **(A)** A tSNE plot of all cells (n = 23,261 cells) in an scRNA-seq data set for NSCLC is shown color-coded according to immune cell or nonimmune cell types. **(B)** A tSNE plot of all immune cells (n = 13,431 cells) is shown color-coded according to cell subsets. **(C)** Normalized expression of IL-15, IL- 27, IFN-γ, IL-10, IL-1β, M-CSF, IL-16, IL-21, and TGF-β genes in immune cells is shown on the tSNE plot as in **(B)**. **(D, E)** Dot plots for average expression of the IFN-γ gene in NK cells **(D)** or T cells **(E)** in each sample of normal lung tissue (n = 11) or early-stage (n = 11) or advanced-stage (n = 21) tumor tissue for the data analyzed in Figure 2. These samples include negative status (normal lung tissue (n = 6) or early-stage (n = 6) or advanced-stage (n = 12) tumor tissue) and positive status (normal lung tissue (n = 4) or early-stage (n = 4) or advanced-stage (n = 6) tumor tissue) for activating *EGFR* mutations. The mean ± SEM values are indicated.


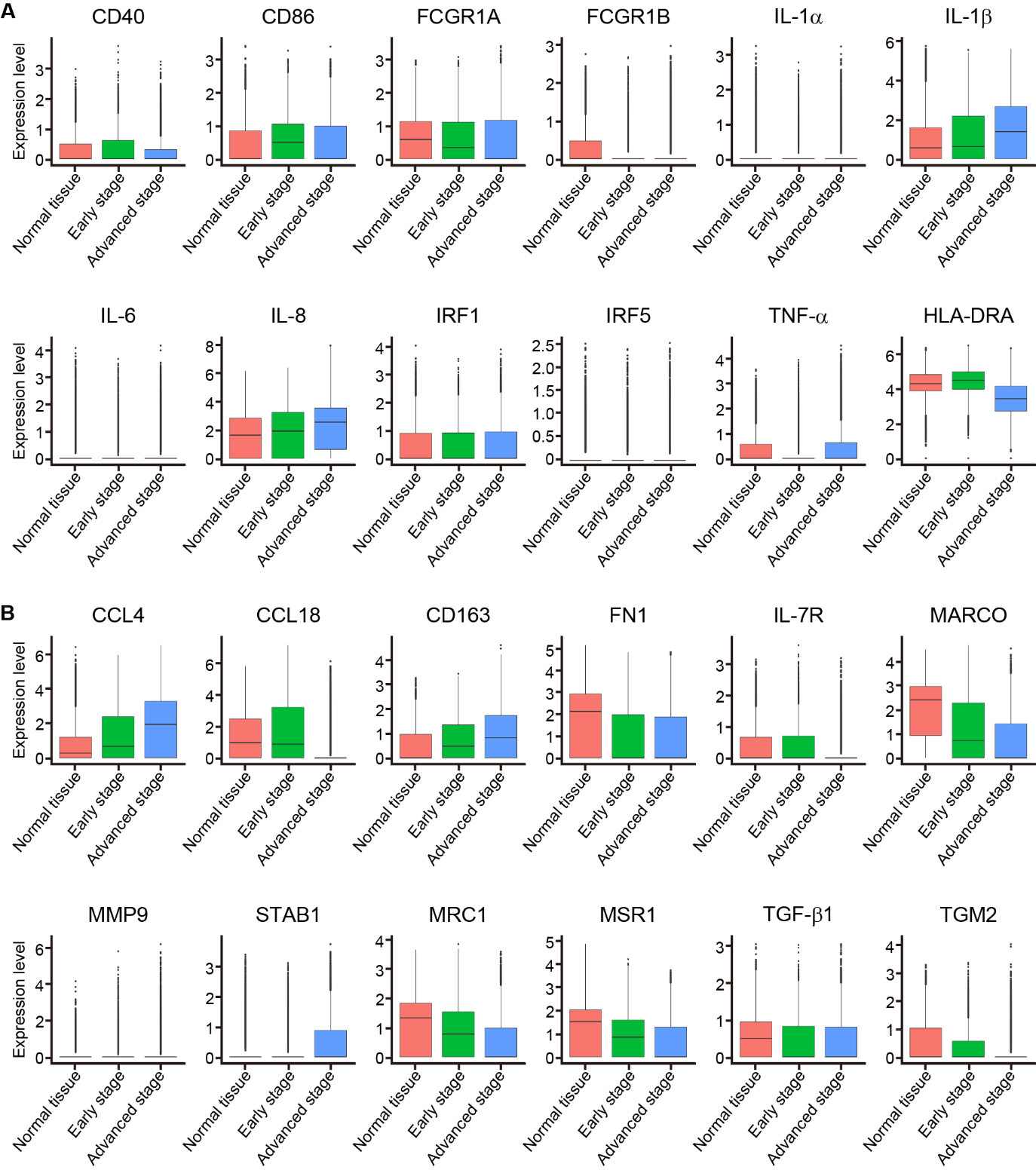


**Supplementary Figure 3. Macrophage phenotype in the TME of NSCLC. M1-phenotype of macrophages in the TME of advanced-stage NSCLC.** Normalized expression of M1 **(A)** or M2 **(B)** signature genes is shown for all myeloid (CD68-expressing) cells derived from normal lung tissue or early-stage or advanced-stage NSCLC tissue of the data set analyzed in Figure 2.


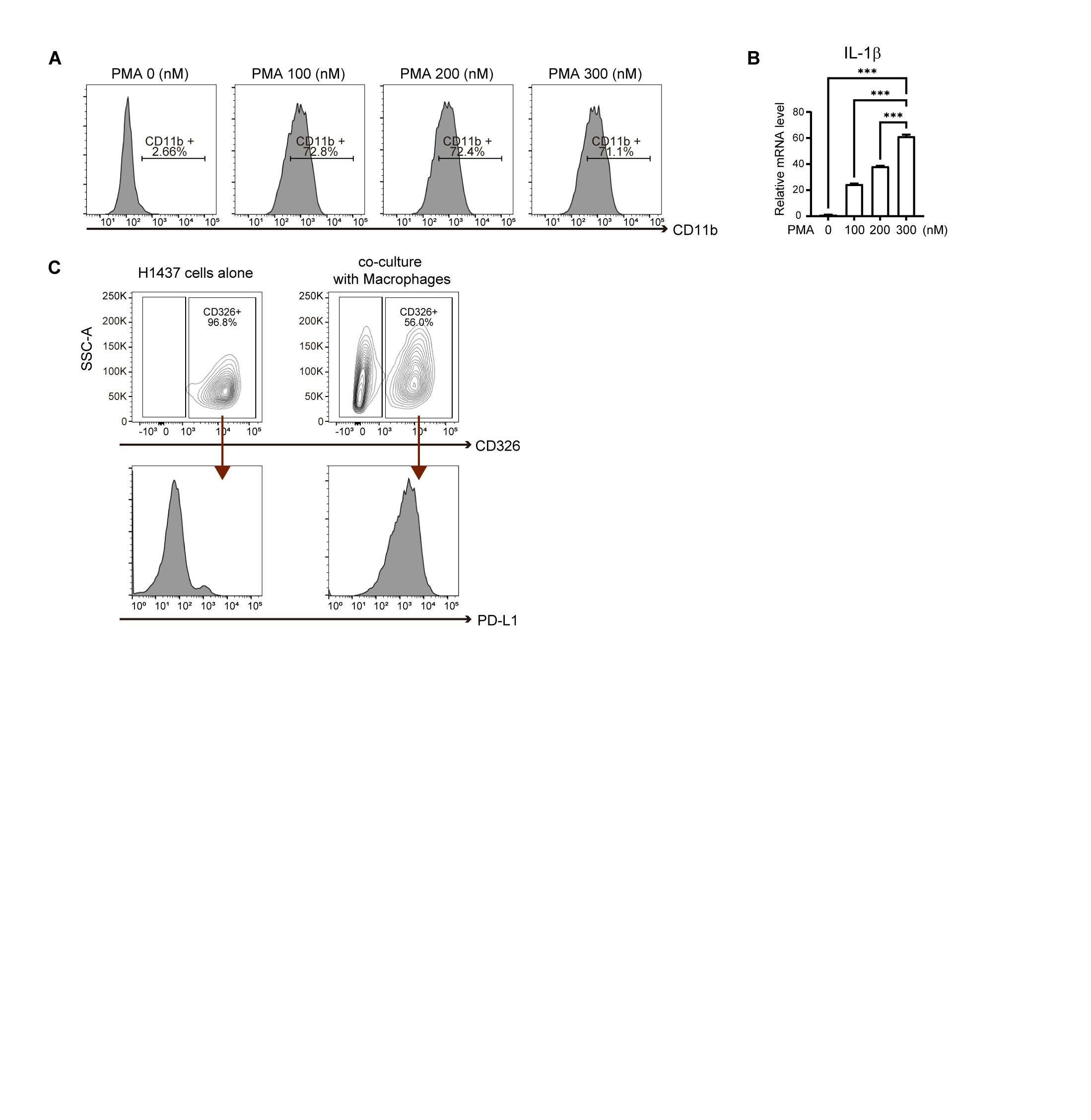


**Supplementary Figure 4. Surface expression of PD-L1 in H1437 cells after co-culture with macrophages. (A)** Representative flow cytometric traces for surface CD11b expression in THP-1 cells were stimulated with stimulated with 100, 200, or 300 nM PMA for 48 h, and then incubated in fresh RPMI 1640 medium for 72 h. **(B)** RT-qPCR analysis of IL-1β mRNA abundance in THP-1 cells were stimulated with 100, 200, or 300 nM PMA. Data are expressed relative to the value for control cells (0 nM PMA) are means + SEM of triplicates from one experiment. **(C)** PD-L1 expression in H1437 cells co-cultured with or without macrophage differentiated by PMA 300 nM stimulation. Representative flow cytometric plots gated on CD326 ^+^ cells, and traces for surface PD-L1 expression in CD326 ^+^ cells.


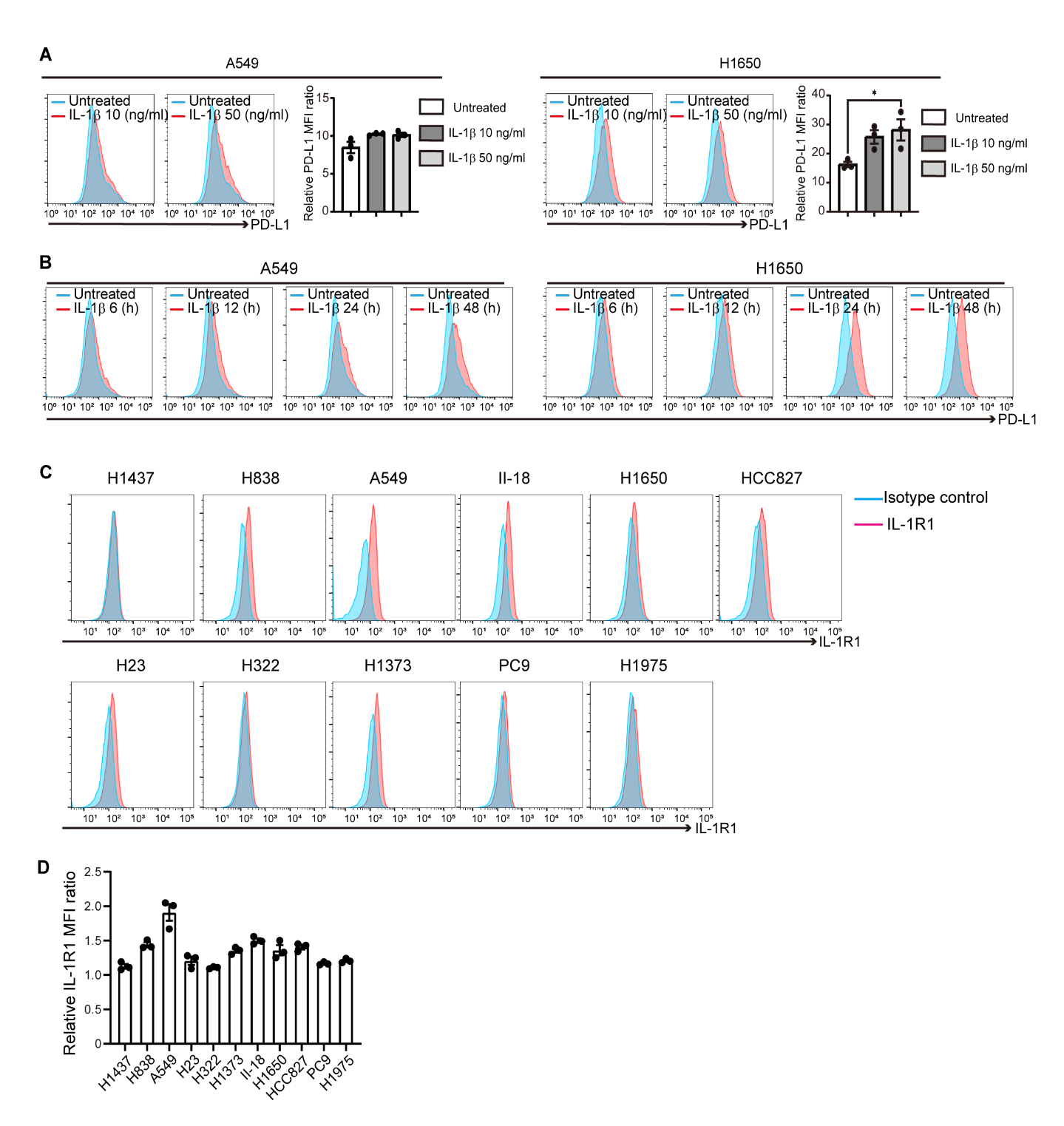


**Supplementary Figure 5. Effect of IL-1β on PD-L1 expression and surface expression of IL-1R1 in NSCLC cell lines. (A)** Flow cytometric analysis of surface PD-L1 expression in A549 and H1650 cells treated with IL-1β at 10 or 50 ng/ml for 24 h. Representative traces and quantitative data are shown, with the latter being expressed as the relative MFI ratio (PD-L1 to isotype control ratio) and presented as means ± SEM from three independent experiments. **(B)** Flow cytometric analysis of surface PD-L1 expression in A549 and H1650 cells treated with IL-1β at 50 ng/ml for 6, 12, 24, or 48 h. **(C)** Representative flow cytometric traces for surface IL-1R1 expression in NSCLC cell lines. **(D)** Relative MFI ratio (IL-1R1 to isotype control ratio) for surface IL-1R1 expression in cell lines as in **(C)**. Data are means ± SEM from three independent experiments. **P* < 0.05 by the Tukey-Kramer test.


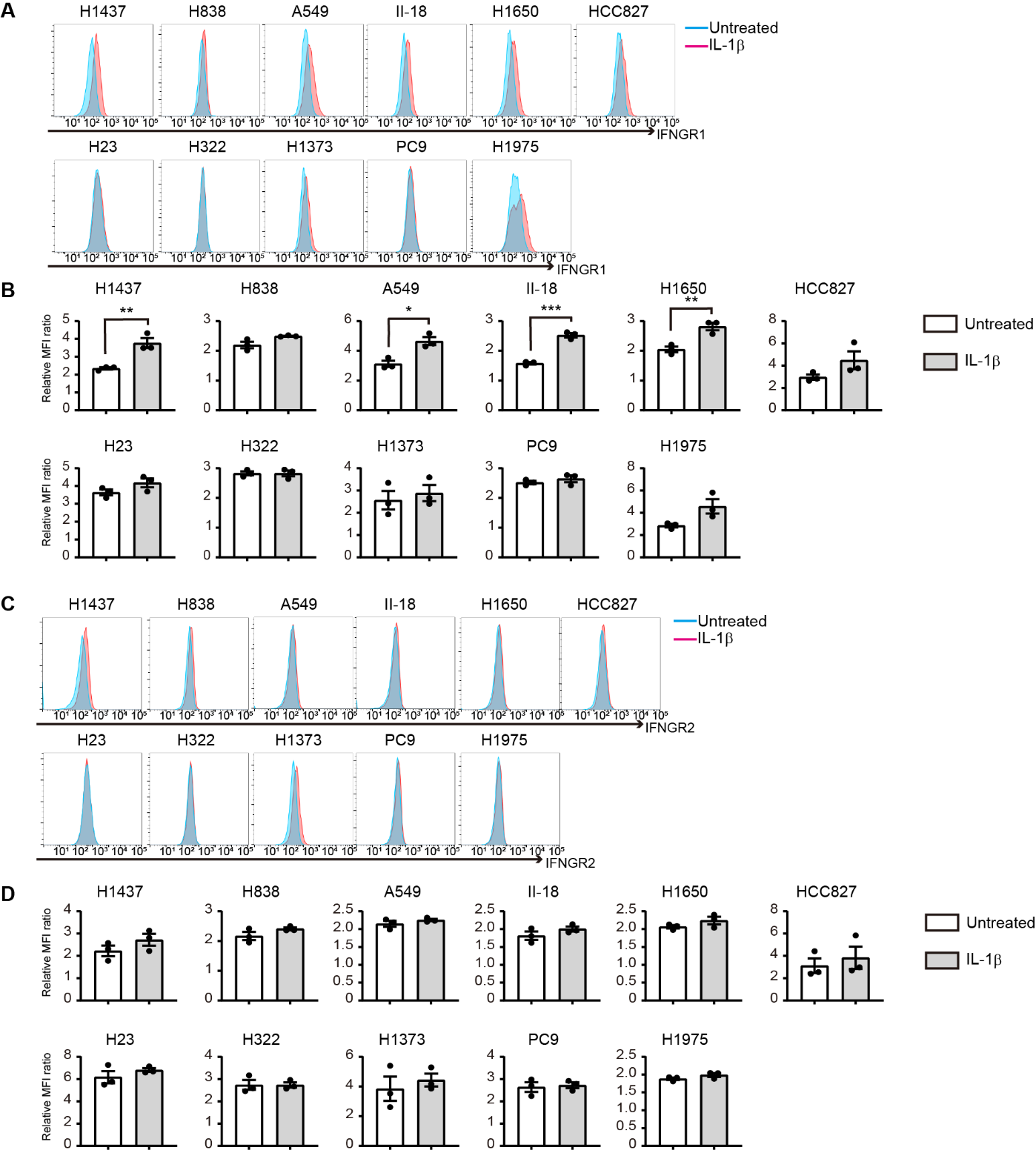


**Supplementary Figure 6. Effects of IL-1β on surface expression of IFNGR1 and IFNGR2 in NSCLC cell lines.** Surface expression of IFNGR1 **(A, B)** or IFNGR2 **(C, D)** in NSCLC cells treated with IL-1β (50 ng/ml) for 12 h was determined by flow cytometry. Representative traces **(A, C)** and quantitative data **(B, D)** are shown. The quantitative data are expressed as the relative MFI ratio (IFNGR1 or IFNGR2 to isotype control ratio) and are means ± SEM from three independent experiments. **P* < 0.05, ***P* < 0.01, ****P* < 0.001 by the Student’s *t* test.


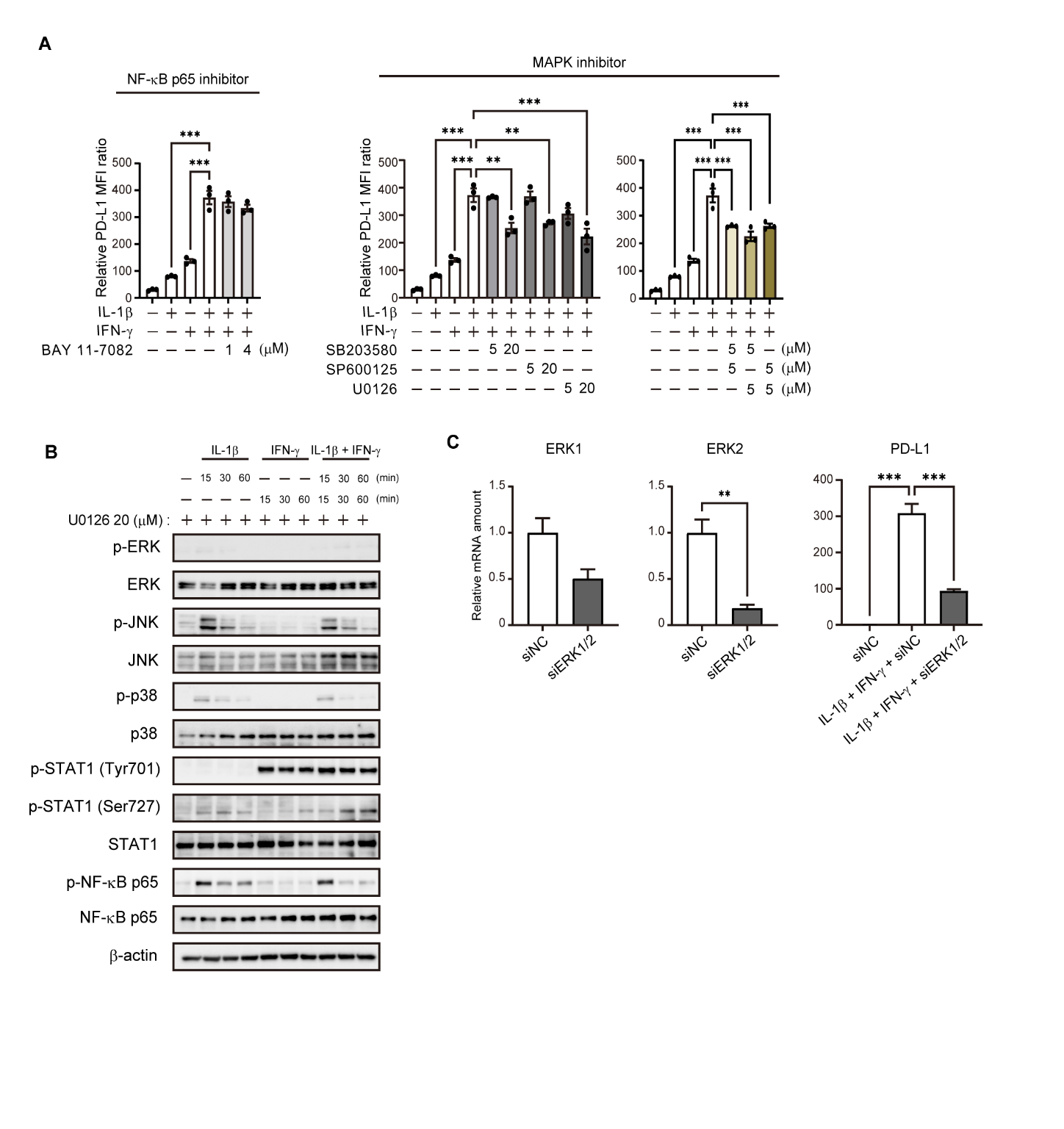


**Supplementary Figure 7. MAPK pathway inhibition attenuates synergistic upregulation of PD-L1 expression by IL-1β and IFN-γ in NSCLC cell lines. (A)** II-18 cells were incubated for 1 h with or without BAY 11-7082 (1 or 4 μM), SB203580 (5 or 20 μM), SP600125 (5 or 20 μM), or U0126 (5 or 20 μM), and then for 24 h in the additional absence or presence of IL-1β (50 ng/ml), IFN-γ (50 ng/ml), or both IL-1β (50 ng/ml) and IFN-γ (50 ng/ml), after which surface expression of PD-L1 was determined by flow cytometry. The quantitative data are expressed as the relative MFI ratio (PD-L1 to isotype control ratio) and presented as means ± SEM from three independent experiments. **(B)** H1437 cells were incubated for 1 h with U0126 (20 μM) and then treated with IL-1β (50 ng/ml), IFN-γ (50 ng/ml), or both IL-1β (50 ng/ml) and IFN-γ (50 ng/ml) for the indicated times, after which cell lysates were subjected to immunoblot analysis with antibodies to total or phosphorylated (p) forms of the MAPKs ERK, JNK, and p38, STAT1, or the p65 subunit of NK-κB. **(C)** H1437 cells were transfected with negative control (NC) or ERK1/2 small interfering RNAs (siRNAs) for 48 h, and subsequently treated with both IL-1β (50 ng/ml) and IFN-γ (50 ng/ml) for 24 h. After which, the cells were subjected to RT-qPCR of relative ERK1, ERK2, or PD-L1 mRNA abundance. Data are means + SEM of triplicates from one experiment and are representative of three independent experiments. ***P* < 0.01, ****P* < 0.001 by the Tukey-Kramer test.
